# Notes on an Unusual Case of Hydatid Cyst

**Published:** 1909-05-22

**Authors:** Wm. H. Smailes


### THE GENERAL PRACTITIONER'S COLUMN.

**[Contributions to this Column are invited, and if accepted will be paid for.]**

### NOTES ON AN UNUSUAL CASE OF HYDATID CYST.

By WM. H. SMAILES, M.B., B.S.Lond.

I HAVE thought the following case worthy of notice because of the comparative rarity of similar cases outside hospital wards, and especially because of the unusual position of the cyst. The patient, a married woman of 46 with several children, came under observation on account of a large tender swelling situated in the upper part of the left thigh directly over the saphenous opening, and extending backwards between the legs, thus causing considerable difficulty in walking.

The history she gave was that 25 years previously, when working in the mill, she "strained" herself when lifting a heavy weight, and a small lump came down in the upper part of the left thigh, apparently causing symptoms of intestinal obstruction. She recovered from this attack by resting in bed, but the lump got gradually bigger, and the symptoms recurred at intervals, the swelling being inflamed and tender on each occasion. As she was very much opposed to operation from the first, she continued to suffer in this way through this long period of years: her opposition to surgical treatment was increased by the doubt cast upon the diagnosis of hernia by some medical men who saw her. On examination in July 1908 there was a large rounded tender swelling in the upper part of the left thigh projecting backwards in the adductor region, and corresponding in size to a cocoanut: it was fixed in the subcutaneous tissue, but was not adherent to either the femur or the skin, and it appeared to be connected to Poupart's ligament by a band of thickened tissue corresponding in position to the crural canal and about the width of the finger. The resemblance at first sight to one of those large femoral hernias which one occasionally sees in hospital patients was most striking, and, as a slight impulse was thought to be obtained with coughing, the diagnosis of femoral hernia seemed justifiable, considering the history of the illness; the swelling could not be reduced, but occasional attacks of peritonitis in the sac would easily explain this. There was a slight sensation of fluctuation imparted to the hands on palpation, but no hydatid fremitus was observed, and the diagnosis of hydatid cyst was not thought of, though the fluctuation suggested the possible presence of a cyst. On careful questioning it was elicited that 22 years previously she had been to the local hospital, and was there advised to have the swelling left severely alone, as any operation would kill her. I suggest that the diagnosis here thought of was aneurysm of the femoral artery: this warning had the unfortunate effect of discouraging the patient still further from undergoing any operation at a later date. As all attempts at diagnosis were eventually proved to be wrong, it may be interesting to consider what other possible causes might account for the condition. No abdominal swelling could be made out in the sheath of the psoas or elsewhere, so a spinal origin was excluded: the hip-joint was freely movable, and there was no shortening of the left leg, so that hip-joint disease was negatived. The femur was quite free, excluding any bony origin for the swelling: a tuberculous abscess of this size would certainly not have existed for so long without bursting. The condition might have been a diffuse lipoma, but its behaviour excluded that.

There was a great temptation to explore the swelling, but this was not allowed, and the condition gradually came to a head and burst, the contents of the swelling proving to be daughter cysts varying in size from a marble to a pea, together with strips of chitinous membrane: the patient then gave consent for the natural opening to be enlarged and the cavity lightly scraped. Considerable shock was present and slight fever, but these symptoms speedily cleared up, and after a slight amount of discharge the sinus formed began to heal from the bottom.

Eight months afterwards the sinus was completely healed and the thigh normal in size: no sign of hernia was present, and there was merely the thickened band in the position of the crural canal: the general health was very good—in fact, she felt better than she had done for years, and was putting on flesh; and there were no signs of secondary deposits in the liver or elsewhere. The fluid inside the daughter cysts was non-albuminous, though there were no hooklets, probably from the great age of the cyst.

A possible explanation of this peculiar situation is that the hernial condition was one of omentum only, and that the cyst developed in the tissue inside the hernial sac: the repeated inflammation caused a natural closure of the crural canal, and cured the hernia: the cyst continued to develop and eventually burst, and a spontaneous cure was brought about: hydatid cysts of the omentum are not unknown, but they rarely occur singly.

